# Clinical analysis of 255 children with multiple serous effusions

**DOI:** 10.1080/07853890.2025.2610884

**Published:** 2026-01-02

**Authors:** Wenwen Jin, Liying Lu, Qiqi Gao, Tong Lou, Wei Hu, Yu Chen, Yu Fang, Zhenlang Lin, Wei Lin

**Affiliations:** The Second Affiliated Hospital and Yuying Children’s Hospital of Wenzhou Medical University, Wenzhou Medical University, Wenzhou, Zhejiang, China

**Keywords:** Children, clinical characteristics, multiple serous effusions

## Abstract

**Background:**

This study aimed to analyze the etiological spectrum, clinical features, and pathological correlates of multiple serous effusions in pediatric patients to inform clinical decision-making.

**Materials and methods:**

A retrospective study was conducted on 255 children diagnosed with multiple serous effusions at the Second Affiliated Hospital and Yuying Children’s Hospital of Wenzhou Medical University between January 1, 2014, and July 1, 2024.

**Results:**

The most common identified causes were pneumonia (*n* = 80, 31.37%), and trauma (*n* = 28, 10.98%). Etiologies demonstrated age and sex-specific patterns. Pneumonia predominated in preschool/school-aged children, and trauma was more common among preschool/school-aged boys. Tumors mainly affected school-aged/adolescent males, while connective tissue diseases (primarily systemic lupus erythematosus) predominated in adolescent females. Pleural effusion was the most common manifestation (*n* = 243, 95.3%), followed by peritoneal (*n* = 187, 73.3%) and pericardial effusions (*n* = 136, 53.3%). The nature and distribution of effusions correlated with the underlying disease. Pneumonia/tumor cases exhibited higher susceptibility to pleural and pericardial effusions, predominantly exudative. Connective tissue disease cases frequently demonstrated involvement of all three serous cavities, predominantly exudative. Cardiac insufficiency cases typically presented with transudative effusions. The triple serous cavity effusion group exhibited significantly lower hemoglobin, serum albumin, calcium, and IgM levels (*p* < 0.05), alongside significantly elevated C-reactive protein, brain natriuretic peptide, prothrombin time, international normalized ratio, and D-dimer levels (*p* < 0.05), indicating more severe disease progression.

**Conclusion:**

The etiology of pediatric multiple serous effusions is diverse and closely linked to patient age and effusion location. The number of affected cavities may serve as an indicator of disease severity.

## 1. Introduction

Multiple serous effusions (MSE) is a complex clinical syndrome characterized by the simultaneous or sequential accumulation of fluid within two or more serous cavities, including the pleural, peritoneal, and pericardial cavities [1,2]. Pediatric MSE is a clinically prevalent condition with significant incidence rates. During the COVID-19 pandemic, MSE was frequently observed in children with multisystem inflammatory syndrome [3,4]. Pediatric MSE exhibits complex and multifactorial etiologies, with distinct patterns of causative factors observed across several age groups [5]. The range of underlying conditions encompasses but is not limited to diverse inflammatory processes, neoplastic diseases (notably leukemia and lymphoma), tuberculosis, connective tissue disorders (including systemic lupus erythematosus and dermatomyositis), familial Mediterranean fever, hepatic cirrhosis, parasitic infections, trauma, and cardiac pathologies [6–8]. Affected children clinically exhibit diverse and nonspecific manifestations, including fever, cough, chest tightness, palpitations, fatigue, abdominal distension, and lower extremities edema. Although these symptoms exhibit a correlation with the anatomical location of effusions, their lack of specificity frequently results in diagnostic confusion with other pediatric conditions, hence increasing the likelihood of misdiagnosis and missed diagnosis [5].

Pediatric MSE, although clinically common and etiologically complex, remains insufficiently studied in terms of their pathogenesis, accurate diagnosis, and optimized treatment strategies, especially regarding comprehensive clinical data analysis. The limited existing literature primarily focuses on etiological investigations, including parasitic diseases and connective tissue disorders [7,9]. Herein, we systematically collected and retrospectively analyzed clinical data from pediatric patients presenting with the most common types of effusions—pleural, peritoneal, and pericardial—to conduct a comprehensive investigation of the common etiologies, clinical features, and underlying mechanisms of pediatric MSE. This study aimed to offer valuable insights for enhancing the diagnosis and management of this condition in clinical practice.

To address this gap, we conducted a retrospective study of 255 pediatric patients diagnosed with MSE at the Second Affiliated Hospital and Yuying Children’s Hospital of Wenzhou Medical University over a decade. The primary objective of this study was to systematically describe the common etiologies and their correlation with age and gender in a large cohort of children. Furthermore, we aimed to investigate the anatomical patterns of effusion distribution and their association with specific underlying diseases. Finally, we sought to explore whether the extent of serous cavity involvement (i.e. the number of cavities affected) could serve as a practical clinical indicator of disease severity. We believe that the findings from this comprehensive analysis will provide valuable insights to guide earlier diagnosis and improved management of this complex condition in children.

## 2. Materials and methods

### 2.1. Study design

We retrospectively collected clinical data of children diagnosed with MSE at the Second Affiliated Hospital and Yuying Children’s Hospital of Wenzhou Medical University between January 1, 2014, and July 1, 2024, from the electronic medical record management system. The inclusion criteria included (1) patients aged <16 years and (2) patients diagnosed with MSE, confirmed by imaging evidence of fluid accumulation in two or more serous cavities (pleural, pericardial, or peritoneal). The exclusion criteria included (1) patients with effusion involving only a single serous cavity, (2) patients with effusions in locations other than pleural, pericardial, or peritoneal spaces, and (3) patients with incomplete medical records. The study protocol adhered to the principles outlined in the Declaration of Helsinki and was approved by the ethics committee of the Second Affiliated Hospital and Yuying Children’s Hospital of Wenzhou Medical University (approval number: 2024-K-418-01). We confirm that all data were confidential and anonymized. Informed consent requirement was waived by the ethics committee of the Second Affiliated Hospital and Yuying Children’s Hospital of Wenzhou Medical University due to the retrospective nature of the current study.

### 2.2. Statistical analysis

Statistical Package for the Social Sciences (SPSS) software (version 27.0) was utilized for statistical analysis. Qualitative data are expressed as percentages. Intergroup comparisons were performed using the chi-square or Fisher’s exact test. Quantitative data are presented as the median and quartiles [P50 (P25, P75)]. Independent samples *t* tests were utilized for intergroup comparisons for normally distributed quantitative data, while the independent samples Mann–Whitney *U* test was utilized for non-normally distributed data. *p <* 0.05 was considered statistically significant.

## 3. Results

### 3.1. Baseline characteristics of patients and clinical outcomes

This study included 255 cases, with 147 (57.6%) males and 108 (42.4%) females. Of the 255 cases, 125 were rural children, and 130 were from dispersed urban areas. The age distribution was categorized as follows: infancy (birth to <3 years), 56 cases (22.0%); preschool age (3 to <6 years), 53 cases (20.8%); school age (6 to <12 years), 82 cases (32.2%); and adolescence (12 to <16 years), 64 cases (25.1%). Their median age was 7 years. Cases occurred throughout all seasons, indicating higher incidence in summer (*n* = 70, 27.5%) and winter (*n* = 72, 28.2%). The survival rate was 89.8% (*n* = 229), with 26 cases (10.2%) deaths. Among deceased cases, the distribution of effusion patterns was as follows: pleural combined with peritoneal effusions, 11 cases (42.3%); pleural with pericardial effusions, 8 cases (30.8%); peritoneal with pericardial effusions, 2 cases (7.7%); and triple serous cavity involvement, 5 cases (19.2%). Table 1 presents the baseline characteristics.

**Table 1.** Baseline characteristics of 255 pediatric patients with MSE.

Characteristic	No. (%)
Gender	
Male	147 (57.6)
Female	108 (42.4)
Age, year	7 (3–12)
Season of onset	
Spring (March–May)	60 (23.5)
Summer (June–August)	70 (27.5)
Autumn (September–November)	53 (20.8)
Winter (November–February of the following year)	72 (28.2)
Place of residence	
Rural	125 (49.0)
Urban	130 (51.0)
Outcome	
Survived	229 (89.8)
Deceased	26 (10.2)

Data are presented as median (P25, P75) or *n* (%).

### 3.2. The etiological composition of pediatric MSE cases and their age and gender distribution

Among the pediatric cases with MSE, the primary diagnoses at admission were pneumonia, 80 cases (31.37%), and trauma, 28 cases (10.98%). Pneumonia predominantly affected preschool-aged (*n* = 21, 26.25%) and school-aged children (*n* = 34, 42.5%), with no significant gender predilection. Trauma was more common among preschool-aged (*n* = 13, 46.43%) and school-aged children (*n* = 7, 25.00%), with a male predominance (*n* = 19, 67.86%). Neoplasms exhibited a predilection for school-aged (*n* = 12, 52.17%) and adolescent cases (*n* = 9, 39.13%), with mediastinal tumors being the most common, with a significant male predominance (*n* = 16, 69.57%). Connective tissue diseases, particularly systemic lupus erythematosus, exhibited a strong association with adolescence (*n* = 13, 68.42%) and female gender (*n* = 13, 68.42%). Glomerulonephritis primarily affected school-aged (*n* = 3, 60%) and adolescent children (*n* = 2, 40%), with a female predominance (*n* = 4, 80%). Cardiac insufficiency cases exhibited no significant age-specific distribution but exhibited a male predominance (*n* = 6, 75%). Furthermore, congenital heart disease (CHD) and postoperative CHD were more commonly observed in infants and/or preschool-aged children, while tuberculosis, pancreatitis, and nephrotic syndrome were more common in school-aged and/or adolescent children, all exhibiting male predominance. Table 2 presents the comprehensive data.

**Table 2.** Clinical diagnoses of 255 pediatric patients with MSE.

Clinical diagnoses	Age group				Gender		Total
	Infancy and toddlerhood (0 to <3 years)	Preschool age (3 to <6 years)	School age (6 to <12 years)	Adolescence (12 to <16 years)	Male	Female	
Pneumonia	15(18.75)	21(26.25)	34(42.50)	10(12.5)	37(46.25)	43(53.75)	80(31.37)
Neoplasms	0(0)	2(8.70)	12(52.17)	9(39.13)	16(69.57)	7(30.43)	23(9.02)
Tuberculosis	0(0)	0(0)	1(25.00)	3(75.00)	3(75.00)	1(25.00)	4(1.57)
Pancreatitis	0(0)	0(0)	5(62.50)	3(37.50)	7(87.50)	1(12.50)	8(3.14)
Connective tissue diseases	1(5.26)	2(10.53)	3(15.79)	13(68.42)	6(31.58)	13(68.42)	19(7.45)
Cardiac insufficiency	3(37.50)	1(12.50)	0(0)	4(50.00)	6(75.00)	2(25.00)	8(3.14)
CHD	7(87.50)	1(12.50)	0(0)	0(0)	6(75.00)	2(25.00)	8(3.14)
Postoperative CHD	1(25.00)	2(50.00)	0(0)	1(25.00)	2(50.00)	2(50.00)	4(1.57)
Nephrotic syndrome	1(9.09)	1(9.09)	5(45.45)	4(36.36)	8(72.73)	3(27.27)	11(4.31)
Glomerulonephritis	0(0)	0(0)	3(60.00)	2(40.00)	1(20.00)	4(80.00)	5(1.96)
Hepatitis	2(100)	0(0)	0(0)	0(0)	1(50.00)	1(50.00)	2(0.78)
Trauma	4(14.29)	13(46.43)	7(25.00)	4(14.29)	19(67.86)	9(32.14)	28(10.98)
Unknown etiology	11(61.1)	1(5.56)	4(22.22)	2(11.11)	13(72.22)	5(27.78)	18(7.06)
Other	11(29.73)	9(24.32)	8(21.62)	9(24.32)	22(59.46)	15(40.54)	37(14.51)

Data are presented as *n* (%).

### 3.3. The distribution characteristics of MSE caused by different etiologies

**Figure 1.**
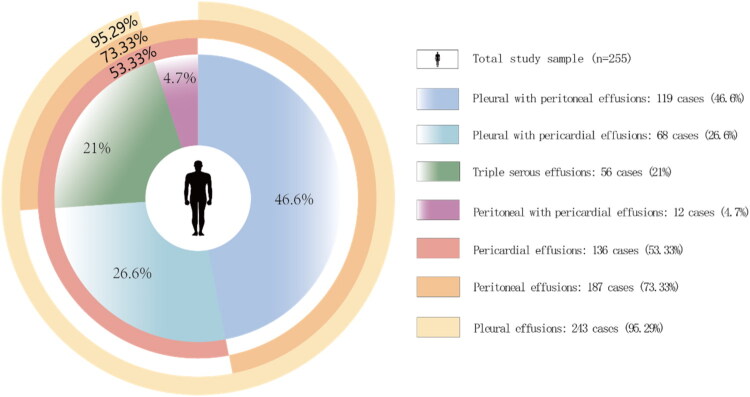
Distribution of effusion sites in 255 pediatric patients with MSE. The outer ring illustrates the percentage distribution of patients with pleural (yellow), peritoneal (orange), or pericardial (pink) effusions relative to the total cohort. The inner ring illustrates the proportional distribution of patients with four distinct patterns of MSE: pleural and peritoneal (blue), pleural and pericardial (cyan), triple serous cavity involvement (green), and peritoneal and pericardial (purple) effusions.

**Figure 2.**
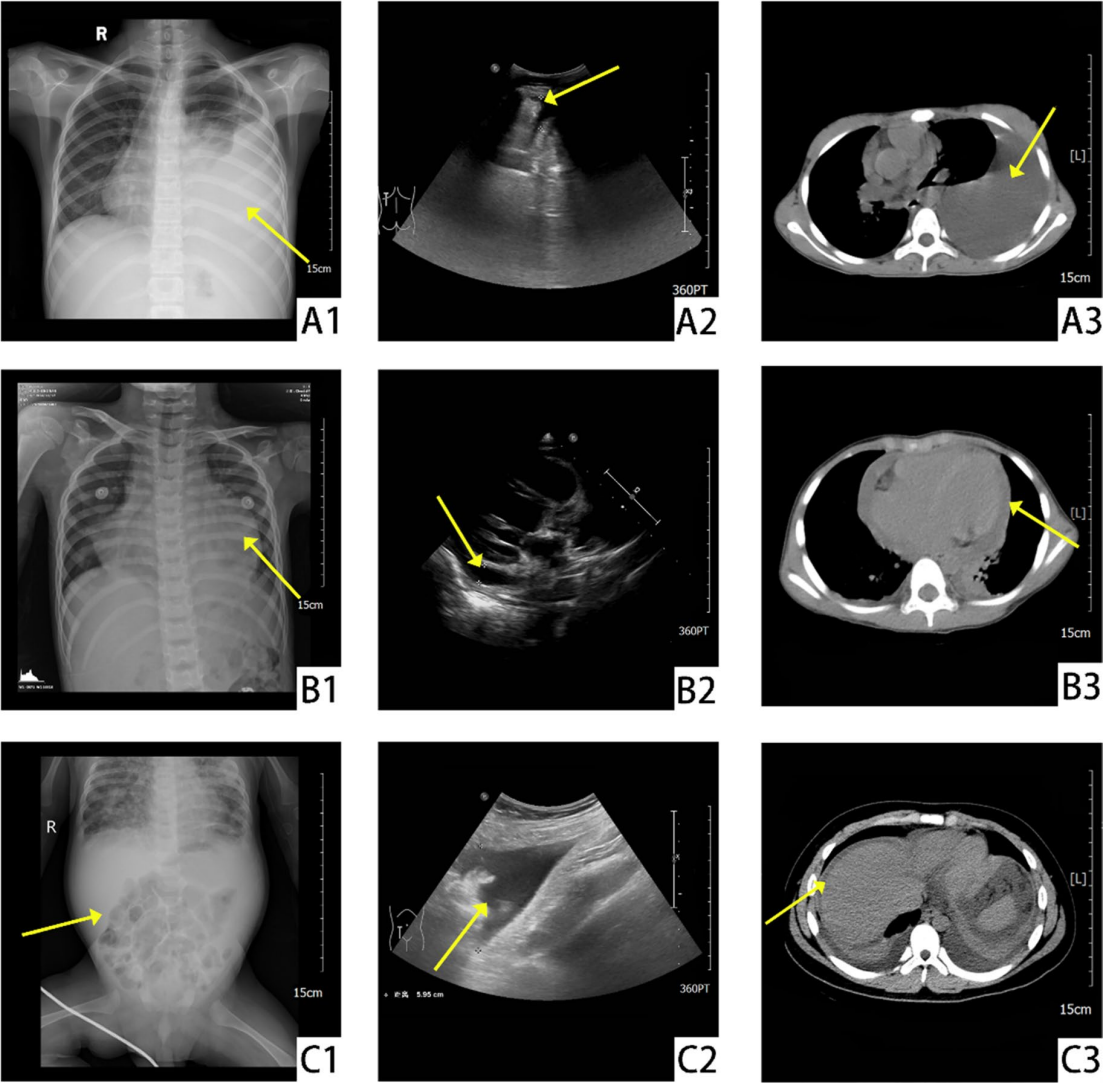
Representative imaging findings in pediatric patients with MSE (radiograph, ultrasonography, computed tomography [CT]). (A1–A3) illustrate characteristic imaging features of massive left pleural effusion on radiographs, ultrasonography, and CT, respectively. (B1–B3) illustrate typical pericardial effusion manifestations across the three imaging modalities. (C1–C3) illustrate radiographic findings of peritoneal effusion visualized by each technique.

Among the 255 cases, pleural effusion was observed in 243 cases (95.3%), peritoneal effusion in 187 cases (73.3%), and pericardial effusion in 136 cases (53.3%) (Figure 1). Cases with pneumonia exhibited a higher incidence of pleural (*n* = 78, 97.5%) and pericardial effusions (*n* = 56, 70%), a pattern similarly observed among those with tumors (pleural: 95.65%, *n* = 22; pericardial: 73.91%, *n* = 17), with approximately 20% of cases in both groups exhibiting triple serous cavity involvement. Children with connective tissue diseases frequently developed effusions in all three cavities (pleural: 89.47%, *n* = 17; peritoneal: 78.95%, *n* = 15; pericardial: 68.42%, *n* = 13), and a significant proportion (36.84%, *n* = 7) presented with triple cavity effusions. Cases with tuberculosis, pancreatitis, nephrotic syndrome, glomerulonephritis, or trauma predominantly developed pleural and peritoneal effusions. However, those with cardiac insufficiency, CHD, or postoperative CHD were more susceptible to pericardial effusion (Table 3). Figure 2 depicts the imaging findings of characteristic effusions.

**Table 3.** Association between etiology and effusion sites in 255 pediatric patients with MSE.

Etiology	Pleural effusion	Peritoneal effusion	Pericardial effusion	Triple serous cavity effusion	Total
Pneumonia	78(97.50)	42(52.50)	56(70.00)	16(20.00)	80(31.37)
Neoplasms	22(95.65)	12(52.17)	17(73.91)	5(21.74)	23(9.02)
Tuberculosis	4(100.00)	3(75.00)	1(25.00)	0(0)	4(1.57)
Pancreatitis	8(100.00)	8(100.00)	1(12.50)	1(12.50)	8(3.14)
Connective tissue diseases	17(89.47)	15(78.95)	13(68.42)	7(36.84)	19(7.45)
Cardiac insufficiency	5(62.50)	8(100.00)	7(87.50)	4(50.00)	8(3.14)
CHD	6(75.00)	7(87.50)	4(50.00)	1(12.50)	8(3.14)
Postoperative CHD	4(100.00)	1(25.00)	3(75.00)	0(0)	4(1.57)
Nephrotic syndrome	11(100.00)	11(100.00)	0(0)	0(0)	11(4.31)
Glomerulonephritis	5(100.00)	5(100.00)	3(60.00)	3(60.00)	5(1.96)
Hepatitis	2(100.00)	2(100.00)	1(100.00)	1(100.00)	2(0.78)
Trauma	28(100.00)	26(92.86)	4(14.29)	2(7.14)	28(10.98)
Unknown etiology	17(94.44)	15(83.33)	8(44.44)	4(22.22)	18(7.06)
Other	36(97.30)	32(86.49)	18(48.65)	12(32.43)	37(14.51)

Data are presented as *n* (%).

### 3.4. Laboratory analysis of serous cavity effusion: distribution, nature and association with etiology

**Figure 3.**
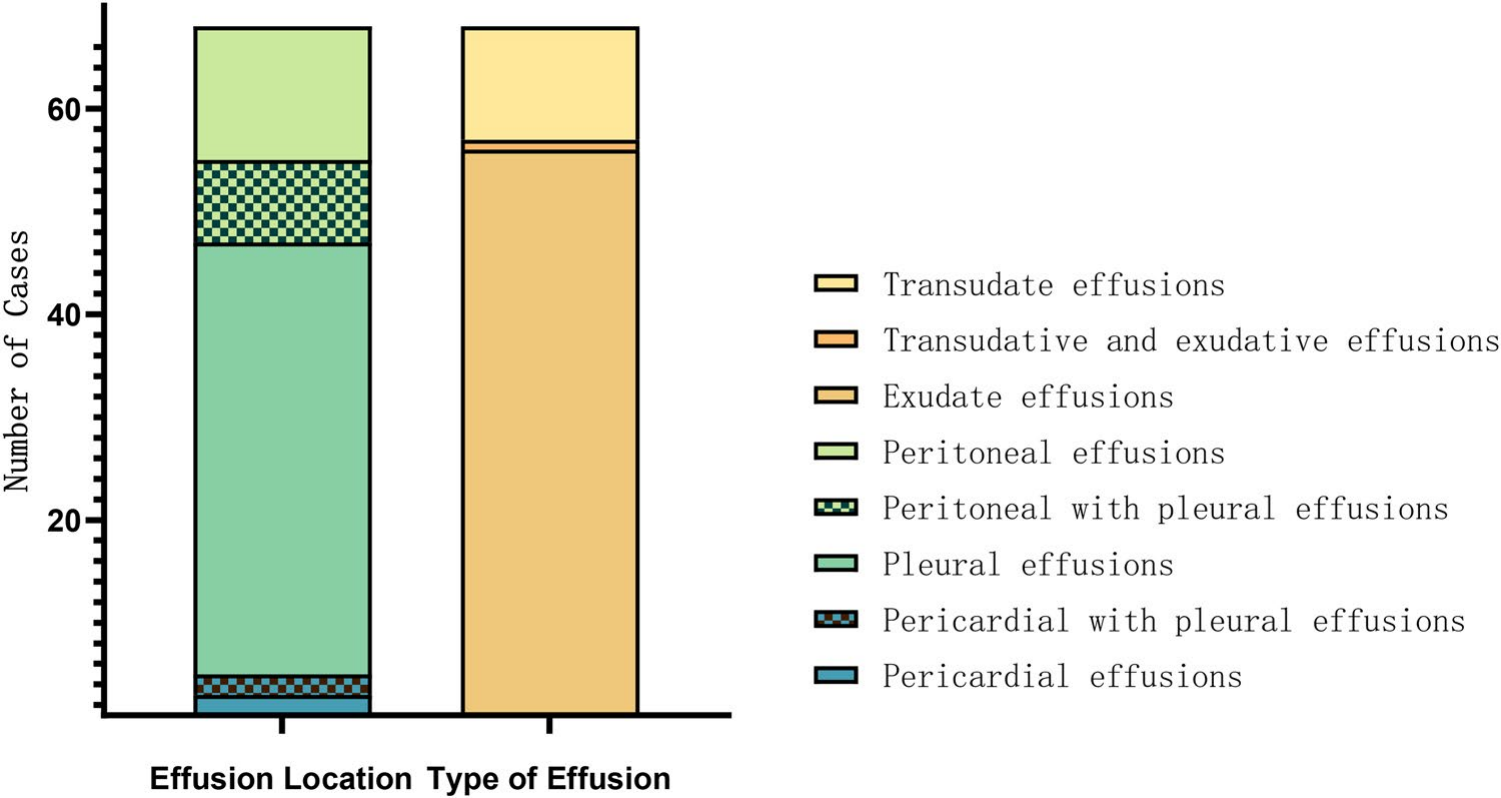
Distribution of effusion locations and types in 68 pediatric cases with MSE. Among the 68 pediatric patients who underwent effusion analysis, the effusion sites included pericardial effusion (2 cases), pericardial and pleural effusion (2 cases), pleural effusion (42 cases), pleural and peritoneal effusion (8 cases), and peritoneal effusion (13 cases). Biochemical analysis revealed exudative effusions in 56 cases (82.4%), transudative effusions in 11 cases (16.2%), and mixed exudative-transudative characteristics in 1 case (1.5%).

**Figure 4.**
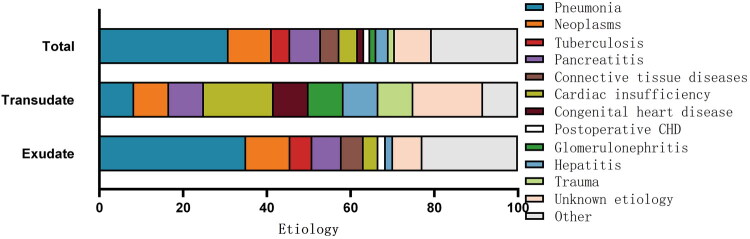
Etiological distribution and types of effusions in 68 pediatric patients with MSE. Biochemical analysis of 68 pediatric cases revealed 57 (83.8%) exudates and 12 (17.6%) transudates, including one discordant (transudative pleural effusion with concomitant exudative ascites). The etiological distribution revealed the following: pneumonia (*n* = 21; 20 exudates and 1 transudate), neoplasms (*n* = 7; 6 exudates and 1 transudate), tuberculosis (*n* = 3; all exudates), pancreatitis (*n* = 5; 4 exudates and 1 transudate), connective tissue diseases (*n* = 3; all exudates), cardiac insufficiency (*n* = 3; 2 exudates and 1 transudate), CHD (*n* = 1; transudate), postcardiac surgery (*n* = 1; exudate), glomerulonephritis (*n* = 1; transudate), hepatitis (*n* = 2; 1 exudate and 1 transudate in distinct cavities), and trauma (*n* = 1; transudate).

Samples were collected from 68 cases for fluid analysis, which revealed pleural effusion in 52 cases (76.47%), peritoneal effusion in 21 cases (30.88%), and pericardial effusion in 5 cases (7.35%). Laboratory investigation revealed exudative effusions in 57 cases (83.82%) and transudative effusions in 12 cases (17.65%) (Figure 3). Among these 68 cases, the most common etiologies were pneumonia, 21 cases (30.88%), and neoplasms, 7 cases (10.29%). Both conditions predominantly exhibited exudative characteristics. Additionally, all 3 cases with tuberculosis and connective tissue diseases exhibited exudative effusions. Of the 5 cases with pancreatitis, 4 (80%) presented with exudates. However, among 3 cases with cardiac insufficiency, 2 exhibited transudative effusions (Figure 4).

### 3.5. Comparison of laboratory test results between cases in the two-cavity group and the triple-cavity group

The study population of 255 pediatric cases was categorized into two groups: those with effusions in two serous cavities (199 cases) and those with triple serous cavity effusions (56 cases), based on anatomical involvement. All participants underwent a complete laboratory assessment, including hematological parameters, biochemical profiles, and inflammatory markers at admission (Table 4). Hematological analysis revealed neutrophilia and reduced hemoglobin levels in approximately 50% of cases in both groups, with a significant decrease in hemoglobin in the triple-cavity effusion group (*p* = 0.021). Among 227 cases who underwent C-reactive protein (CRP) testing within 24 h of admission (two-cavity: 173; triple-cavity: 54), elevated levels were detected in 96 cases (55.5%) and 39 cases (72.2%), respectively, with significantly higher CRP concentrations in the triple-cavity group (*p* = 0.012). Procalcitonin levels were assessed in 137 cases (two-cavity: 92; triple-cavity: 31), and no statistically significant difference was observed in elevation rates between groups.

**Table 4.** Laboratory examination results upon admission of 255 MSE patients.

Laboratory indicators	Two-cavity group	Triple-cavity group	*P* value
White blood cell count(×10^9^/L)	10.75 (6.92–15.75)	10.31 (7.76–13.77)	0.901
Neutrophil count (×10^9^/L)	6.83 (4.33–12.04)	7.00 (4.73–9.96)	0.764
Hemoglobin (g/L)	119.00 (105.00–133.00)	111.50 (101.50–123.75)	0.021
Platelet count (×10^9^/L)	271.00 (161.00–366.00)	276.00 (153.50–343.50)	0.994
CRP (mg/L)	11.43 (1.92–54.79)	31.08 (6.46–83.84)	0.012
Procalcitonin (ng/ml)	0.33 (0.10–1.42)	0.37 (0.10–1.26)	0.837
Alanine aminotransferase (IU/L)	23.00 (13.00–55.50)	29.00 (14.50–84.00)	0.346
Aspartate aminotransferase (IU/L)	44.00 (26.00–84.25)	52.00 (27.50–142.00)	0.147
Lactate dehydrogenase (IU/L)	421.00 (292.50–885.00)	509.00 (313.00–1190.00)	0.248
Total bilirubin (µmol/L)	8.45 (5.18–16.05)	10.30 (5.85–15.90)	0.235
Blood urea nitrogen (mmol/L)	4.18 (3.04–5.74)	4.28 (3.05–7.60)	0.534
Creatinine (µmol/L)	37.35 (29.00–51.00)	44.00 (30.80–65.80)	0.046
Creatine kinase (IU/L)	118.5 (68.50–242.25)	97.50 (43.00–346.50)	0.372
Albumin (g/L)	37.25 (31.60–41.70)	34.00 (28.50–38.40)	0.030
Globulin (g/L)	25.40 (21.13–31.18)	26.40 (21.75–31.15)	0.341
Blood glucose (mmol/L)	5.66 (4.62–6.85)	5.73 (5.09–6.59)	0.864
Serum calcium (mmol/L)	2.19 (2.04–2.31)	2.06 (1.89–2.20)	0.000
Serum sodium (mmol/L)	135.95 (133.08–138.70)	136.20 (133.28–139.35)	0.621
Serum potassium (mmol/L)	4.07 (3.75–4.42)	4.05 (3.65–4.44)	0.849
Troponin-I (ng/mL)	0.01 (0.01–0.04)	0.02 (0.01–0.23)	0.104
B-type natriuretic peptide (pg/mL)	359.00 (91.00–4776.00)	1000 (232.50–17450.00)	0.036
IgA (g/L)	1.62 (1.03–2.43)	1.76 (0.81–2.04)	0.479
IgG (g/L)	8.93 (6.38–12.20)	9.81 (7.75–13.73)	0.103
IgE (IU/mL)	136.75 (40.89–451.78)	244.10 (78.40–707.00)	0.111
IgM (g/L)	1.46 (1.00–1.97)	1.14 (0.79–1.60)	0.038
PT (s)	14.40 (13.50–15.40)	15.40 (14.35–17.25)	0.001
International normalized ratio (INR)	1.15 (1.07–1.24)	1.24 (1.13–1.40)	0.002
Activated partial thromboplastin time (APTT, s)	40.15 (35.60–45.23)	42.00 (34.80–48.60)	0.363
Fibrinogen (FIB, g/L)	3.70 (2.34–5.23)	3.15 (2.20–5.35)	0.300
D-D (μg/mL)	2.27 (1.07–5.62)	5.45 (2.53–10.21)	0.000
CD3 + T lymphocytes (CD3%)	68.82 (63.03–75.59)	66.08 (57.84–72.16)	0.151
CD4 + T helper/inducer cells (CD4%)	34.16 (28.54–39.59)	31.05 (24.21–39.86)	0.156
CD8 + T suppressor/cytotoxic cells (CD8%)	29.34 (23.72–34.63)	27.30 (20.47–31.68)	0.313
CD4/CD8 ratio	1.19 (0.91–1.50)	1.14 (0.78–1.68)	0.891
CD16 + 56+ natural killer cells (NK)%	8.71 (4.41–11.92)	6.89 (4.05–9.90)	0.269
CD19+ B lymphocytes (CD19%)	20.57 (16.55–27.65)	25.12 (16.14–31.30)	0.144

Data are expressed in median (P25, P75). Inter-group comparisons were performed using independent samples *t* test for IgG and Mann–Whitney *U* test for all other parameters.

Among 189 cases evaluated for aspartate aminotransferase, elevated levels were observed in 77 cases with effusions in two serous cavities and 30 cases with triple serous cavity effusions. Of 192 cases who underwent lactate dehydrogenase testing, >80% exhibited levels >246 IU/L, however, no significant intergroup differences were observed. Hypoalbuminemia was common among 243 tested cases, with 119/188 (63.3%) in the two-cavity group versus 47/55 (85.5%) in the triple-cavity group (*p* = 0.030). Significant hypocalcemia was observed in both groups (two-cavity: 101/180 cases, 56.1%; triple-cavity: 42/52 cases, 80.8%), with a significant reduction in the triple-cavity group (*p* < 0.001). Hyponatremia was detected in 113/190 (59.5%) cases and 31/54 (57.4%) cases, respectively, without statistically significant differences. Myocardial injury, evidenced by elevated brain natriuretic peptide levels, occurred in 77.3% of tested cases (two-cavity: 40/55, 72.7%; triple-cavity: 28/33, 84.8%), with significantly higher levels in the triple-cavity group (*p* = 0.036).

Immunoglobulin levels were assessed in 129 cases (with 3 cases without data for immunoglobulin IgA/IgM/IgG data and 28 cases lacking values for IgE). Most cases exhibited normal immunoglobulin levels. However, the triple serous cavity effusion group exhibited significantly lower IgM values than the two-cavity effusion group (*p* = 0.038).

Among 219 cases who underwent coagulation testing (excluding 1 patient with missing international normalized ratio (INR) due to prolonged PT and 23 cases without D-dimer (D-D) measurements), all exhibited normal or slightly elevated prothrombin time (PT) and INR, with significantly higher values in the triple-cavity effusion group (*p* = 0.001 and *p* = 0.002, respectively). Activated partial thromboplastin time and fibrinogen levels were within normal or slightly elevated ranges in both groups, with statistically non-significant differences. Elevated D-D levels were observed in 91.32% of the (two-cavity: 133/148, 89.86%; triple-cavity: 46/48, 95.83%), with significantly greater elevation in the triple-cavity group (*p* < 0.001), indicating clinical relevance.

T-cell subset evaluation was performed in 94 cases, and it revealed normal total T-lymphocyte percentages and T-helper/inducer cell proportions in >50% (70/94 and 56/94) of the cases. Elevated T-suppressor/cytotoxic cell percentages were observed in 46.8% (44/94) of cases without intergroup statistically significant differences. Within the triple serous cavity effusion group, 19 of 31 (61.3%) cases exhibited natural killer cell counts below the lower normal limit, though this finding was statistically non-significant when compared with the double cavity effusion group. Notably, 58 of 94 cases (61.7%) exhibited elevated B-lymphocyte proportions, with non-significant differences between the two groups.

## 4. Discussion

Pediatric MSE, though relatively common, remains insufficiently studied, with no established diagnostic or therapeutic guidelines to date. Herein, we conducted a retrospective analysis of clinical data from pediatric patients with MSE admitted to a large tertiary care hospital in China over a 10-year period. The results indicated no significant age-specific differences in disease incidence; however, a slight male predominance was observed with a male-to-female ratio of 1.36:1, implying potential gender-based susceptibility possibly attributed to increased physical activity and consequent higher trauma exposure in male children [10]. No definitive seasonal trend was established; nevertheless, marginally higher case numbers were observed during summer and winter compared to spring and autumn. Most patients exhibited positive outcomes, with mortality cases predominantly occurring among those presenting with combined pleural–peritoneal effusions or pleural–pericardial effusions.

The etiological spectrum of pediatric MSE exhibits significant complexity and variation. Current data indicate that its pathogenesis is primarily associated with infectious diseases (pneumonia, tuberculosis, and parasitic infections), trauma, neoplasms, and connective tissue disorders [5,9,11]. Our findings validate the existing literature [5], identifying pneumonia and trauma as the predominant etiologies. The etiology exhibits significant age-dependent patterns: pneumonia and trauma predominated in preschool- and school-aged children, while neoplastic causes (particularly mediastinal tumors) become increasingly relevant during school age and adolescence. Connective tissue diseases exhibited a predilection for adolescent females, consistent with the findings of previous studies [12]. Consequently, patient age is a critical diagnostic determinant when investigating MSE etiology.

During disease progression, the pathophysiological imbalance among altered vascular permeability, increased capillary venous pressure, and compromised lymphatic drainage results in abnormal fluid accumulation in serous cavities, resulting in protein-poor transudates or inflammatory exudates [13]. This dichotomy in fluid characteristics reveals unique etiological correlations, with transudates primarily observed in congestive heart failure and hypoalbuminemia states, while exudates may arise from diverse pathological conditions [14]. This study revealed that infectious diseases (pneumonia and tuberculosis), neoplasms, and connective tissue disorders predominantly manifested with exudative effusions, while cardiovascular diseases (CHD and postoperative cases), renal disorders (nephrotic syndrome), and trauma primarily presented with transudative MSE. Furthermore, MSE of distinct etiologies exhibits distinctive anatomical distribution patterns: Pneumonia caused by *Mycoplasma pneumoniae* primarily presents with pleural-pericardial effusions, tuberculous MSE typically presents with pleuro-peritoneal involvement, while systemic lupus erythematosus often demonstrates pleural-pericardial or triple serous cavity effusions [15–17]. Consistent with the findings of previous studies, our study identified pleural-pericardial distribution as the predominant pattern in pneumonia-associated cases. Mediastinal tumors were the predominant neoplastic etiology, demonstrating a preferential involvement of pleural and pericardial cavities, perhaps due to their anatomical proximity and direct compressive or invasive effects on adjacent serous membranes. Pancreatitis, nephrotic syndrome, glomerulonephritis, and hepatitis cases primarily involved pleural and peritoneal cavities, potentially indicating anatomical contiguity with pathological foci, while traumatic cases exhibited predominant pleuro-peritoneal effusions. The anatomical distribution of serous effusions has significant diagnostic implications, necessitating strict adherence to the ‘localization-etiology’ correlation principle in clinical practice, where symptomatic management must be accompanied by systematic treatment of underlying pathologies.

This study categorized enrolled patients into two-cavity and triple-cavity groups based on the number of affected body cavities to clarify the correlation between the extent of serosal involvement and clinical severity. Comparative analyses indicated that the triple-cavity group exhibited significantly lower hemoglobin, serum albumin, serum calcium, and IgM levels, with markedly elevated CRP, serum creatinine, brain natriuretic peptide, PT, INR, and d–d levels compared to the two-cavity group. Given the exploratory nature of these comparisons, the observed associations between the number of involved serosal cavities and distinct laboratory abnormalities should be interpreted with caution. Nevertheless, the potential link these associations suggest to disease severity is consistent with the earlier report by Andrejevic et al. [18], a finding that requires validation in subsequent prospective, multi-center studies with larger sample sizes. In pediatric patients with MSE, the number of affected serous cavities serves as an important clinical indicator for assessing disease severity. As a direct reflection of severity, this parameter also helps identify patients at risk of poor prognosis. This quantitative approach reflects underlying pathophysiological progression patterns and demonstrates practical advantages in clinical implementation, warranting further investigation into its potential utility as a severity stratification tool.

Pediatric MSE is a clinically significant pathological condition whose etiological mechanisms and clinical symptoms remain inadequately understood. This single-center retrospective study examined decadal clinical data to thoroughly delineate the clinical features of affected children, demonstrating that the etiology of pediatric MSE exhibits distinct correlations with patient age and anatomical distribution of effusions, while the involved serosal cavities maintain consistent topographical relationships with primary disease sites. The extent of serosal involvement may act as a potential marker of disease severity.

This study has some limitations, including a relatively constrained sample size, its retrospective design, and incomplete effusion analysis data resulting from clinical practice. Furthermore, our statistical analysis involved multiple statistical comparisons without formal correction for multiplicity. As a result, some of the significant associations we observed, particularly for certain laboratory parameters, should be interpreted as exploratory and may be susceptible to chance findings. Therefore, further investigation through prospective multicenter studies with larger cohorts and a priori hypotheses is warranted to validate these preliminary findings and confirm the identified relationships.

The etiological spectrum of pediatric MSE demonstrates considerable complexity and variability, with patient age serving as a critical diagnostic indicator in elucidating etiology. The anatomical distribution of serous effusions carries significant diagnostic implications. Pneumonia and trauma are the primary etiological factors. Furthermore, the extent of serous cavity involvement may have a positive correlation with disease severity.

## Data Availability

The datasets used and/or analyzed during the current study are available from the corresponding authors on reasonable request.

## Abbreviations

APTT: Activated partial thromboplastin time
CD: Cluster of differentiation
CHD: Congenital heart disease
CRP: C-reactive protein
CT: Computed tomography
D-D: D-dimer
FIB: Fibrinogen
Ig: Immunoglobulin
IgA: Immunoglobulin A
IgE: Immunoglobulin E
IgG: Immunoglobulin G
IgM: Immunoglobulin M
INR: International normalized ratio
MSE: Multiple serous effusions
NK: Natural killer cells
PT: Prothrombin time
